# *trans*-Carbonyl­chloridobis(tri-*o*-tolyl­phosphane-κ*P*)rhodium(I)

**DOI:** 10.1107/S1600536811045715

**Published:** 2011-11-05

**Authors:** Stefan Warsink, Renier Koen, Andreas Roodt

**Affiliations:** a Department of Chemistry, University of the Free State, PO Box 339, Bloemfontein 9300, South Africa

## Abstract

In the title compound, [RhCl(C_21_H_21_P)_2_(CO)], the coordination geometry around the Rh^I^ atom is slightly distorted square-planar with the phosphane ligands in *trans* positions with respect to each other. The chloride and carbonyl ligands show positional disorder, and the Rh^I^ atom lies on a center of inversion. The effective cone angle Θ_E_ for the title compound is 169.0 (3)°. There are no significant inter­molecular inter­actions.

## Related literature

For background information, see: Angoletta (1959 ▶); Vaska & Di Luzio (1961 ▶); For a review of related compounds, see: Roodt *et al.* (2003 ▶). For related structures, see: Meijboom *et al.* (2005 ▶); Otto *et al.* (1999 ▶). 
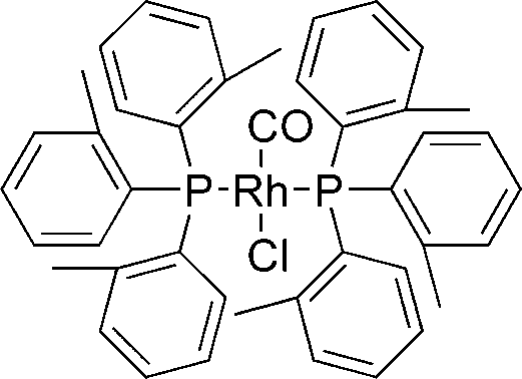


## Experimental

### 

#### Crystal data

[RhCl(C_21_H_21_P)_2_(CO)]
*M* *_r_* = 775.07
Monoclinic, 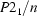

*a* = 10.6440 (14) Å
*b* = 10.9464 (15) Å
*c* = 15.605 (2) Å
β = 93.102 (5)°
*V* = 1815.5 (4) Å^3^
*Z* = 2
Mo *K*α radiation
μ = 0.67 mm^−1^
*T* = 100 K
0.30 × 0.17 × 0.12 mm

#### Data collection

Bruker APEXII CCD diffractometer
Absorption correction: multi-scan (*SADABS*; Bruker, 2007 ▶) *T*_min_ = 0.872, *T*_max_ = 0.921
21616 measured reflections
4481 independent reflections
3344 reflections with *I* > 2σ(*I*)
*R*_int_ = 0.078

#### Refinement

*R*[*F* ^2^ > 2σ(*F* ^2^)] = 0.049
*wR*(*F* ^2^) = 0.143
*S* = 1.03
4481 reflections
235 parameters
H-atom parameters constrained
Δρ_max_ = 2.35 e Å^−3^
Δρ_min_ = −1.13 e Å^−3^

### 

Data collection: *APEX2* (Bruker, 2007 ▶); cell refinement: *SAINT-Plus* (Bruker, 2007 ▶); data reduction: *SAINT-Plus* and *XPREP* (Bruker, 2007 ▶); program(s) used to solve structure: *SHELXS97* (Sheldrick, 2008 ▶); program(s) used to refine structure: *SHELXL97* (Sheldrick, 2008 ▶); molecular graphics: *Mercury* (Macrae *et al.*, 2008 ▶); software used to prepare material for publication: *WinGX* (Farrugia, 1999 ▶).

## Supplementary Material

Crystal structure: contains datablock(s) I, global. DOI: 10.1107/S1600536811045715/pv2465sup1.cif

Structure factors: contains datablock(s) I. DOI: 10.1107/S1600536811045715/pv2465Isup2.hkl

Additional supplementary materials:  crystallographic information; 3D view; checkCIF report

## Figures and Tables

**Fig. 1.**
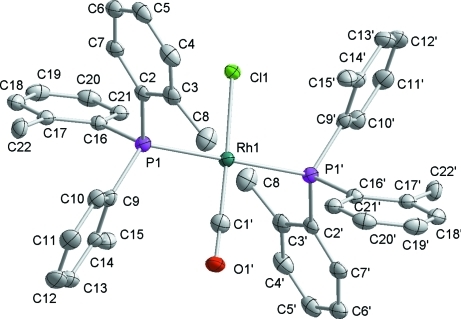
Molecular Structure of (I). Displacement ellipsoids are drawn at the 50% probability level [symmetry code: (i) -x, -y, 1 - z]. H atoms and disordered Chlorido and carbonyl ligands have been omitted for clarity.

## supplementary crystallographic information

### Comment

The complex first synthesized by Angoletta (1959) and later correctly formulated by Vaska (Vaska & Di Luzio, 1961), *trans*-[IrCl(CO)(PPh_3_)_2_] has become known under the name of the latter. This complex and its analogues have been extensively used as catalysts and model compounds (Roodt *et al.*, 2003).

Here we report a rhodium analogue bearing *o*-tolyl substituents on the phosphane ligands (I). As with many of these 'Vaska complexes', compound (I) crystallized with the metal on a crystallographric centre of symmetry, leading to a 50/50 disorder for the chloride and carbonyl ligands. With this report, Vaska complexes bearing all isomers of tritolylphosphane have been described. Compound (I) crystallizes in a slightly distorted square planar geometry with the phosphane ligands in *trans*-position to each other (Fig. 1).

The Rh—C and Rh—Cl bonds do not show large deviations from similar complexes, showing that the steric influence of the *o*-methyl substituents is not so significant as to distort the coordination geometry to a large degree. However, the Rh—P bond is longer than in similar complexes, indicating that the bulky *ortho*-aryl substituents force the phosphane ligands away from the rhodium. A useful indicator for the steric influence of phosphane ligands is the effective cone angle Θ_E_. For compound (I) this angle was found to be 169.0 (3) °, significantly larger than differently substituted triarylphosphanes like tri(*m*-tolyl)-phosphane, for which values of 155° and 160° were reported (Meijboom *et al.*, 2005).

### Experimental

Compound (I) was synthesized by slow addition of 4 equivalents of tri(*o*-tolyl)phosphane to a dimethyl formamide solution of [RhCl(CO)_2_]_2_. After precipitation with ice water and separation of the product, it was recrystallized by slow evaporation from a 1:5 dichloromethane/hexane mixture. Analysis of the compound showed a CO stretching signal in IR at 1972 cm^-1^, and a signal for the phosphane ligands in ^31^P NMR at 25.7 p.p.m. with a J_Rh-P_ of 125.9 Hz. These signals are in good agreement with various other rhodium Vaska's complexes.

### Refinement

The aromatic and methyl H atoms were placed in geometrically idealized positions (C—H = 0.93 and 0.96 Å, respectively) and constrained to ride on their parent atoms with *U*_iso_(H) = 1.2*U*_eq_(C) for aromatic, and *U*_iso_(H) = 1.5*U*_eq_(C) for methyl H-atoms. The highest residual electron density was located 0.87 Å from Rh1 and was essentially meaningless.

### Crystal data

**Table:**

[RhCl(C_21_H_21_P)_2_(CO)]	*F*(000) = 800
*M**_r_* = 775.07	*D*_x_ = 1.418 Mg m^−^^3^
Monoclinic, *P*2_1_/*n*	Mo *K*α radiation, λ = 0.71069 Å
Hall symbol: -P 2yn	Cell parameters from 6713 reflections
*a* = 10.6440 (14) Å	θ = 2.3–28.1°
*b* = 10.9464 (15) Å	µ = 0.67 mm^−^^1^
*c* = 15.605 (2) Å	*T* = 100 K
β = 93.102 (5)°	Cuboid, yellow
*V* = 1815.5 (4) Å^3^	0.30 × 0.17 × 0.12 mm
*Z* = 2	

### Data collection

**Table:**

Bruker X8 APEXII 4K KappaCCD diffractometer	4481 independent reflections
Radiation source: sealed tube	3344 reflections with *I* > 2σ(*I*)
graphite	*R*_int_ = 0.078
Detector resolution: 512 pixels mm^-1^	θ_max_ = 28.3°, θ_min_ = 2.3°
φ and ω scans	*h* = −12→14
Absorption correction: multi-scan (*SADABS*; Bruker, 2007)	*k* = −13→14
*T*_min_ = 0.872, *T*_max_ = 0.921	*l* = −20→19
21616 measured reflections	

### Refinement

**Table:**

Refinement on *F*^2^	Primary atom site location: structure-invariant direct methods
Least-squares matrix: full	Secondary atom site location: difference Fourier map
*R*[*F*^2^ > 2σ(*F*^2^)] = 0.049	Hydrogen site location: inferred from neighbouring sites
*wR*(*F*^2^) = 0.143	H-atom parameters constrained
*S* = 1.03	*w* = 1/[σ^2^(*F*_o_^2^) + (0.0845*P*)^2^ + 0.2003*P*] where *P* = (*F*_o_^2^ + 2*F*_c_^2^)/3
4481 reflections	(Δ/σ)_max_ = 0.001
235 parameters	Δρ_max_ = 2.35 e Å^−^^3^
0 restraints	Δρ_min_ = −1.13 e Å^−^^3^

### Special details

**Table:**

Experimental. The intensity data was collected on a Bruker X8 Apex II 4 K Kappa CCD diffractometer using an exposure time of 60 s/frame. A total of 1376 frames was collected with a frame width of 0.5° covering up to θ=28.27° with 99.5% completeness accomplished.
Geometry. All s.u.'s (except the s.u. in the dihedral angle between two l.s. planes) are estimated using the full covariance matrix. The cell s.u.'s are taken into account individually in the estimation of s.u.'s in distances, angles and torsion angles; correlations between s.u.'s in cell parameters are only used when they are defined by crystal symmetry. An approximate (isotropic) treatment of cell s.u.'s is used for estimating s.u.'s involving l.s. planes.
Refinement. Refinement of *F*^2^ against ALL reflections. The weighted *R*-factor *wR* and goodness of fit *S* are based on *F*^2^, conventional *R*-factors *R* are based on *F*, with *F* set to zero for negative *F*^2^. The threshold expression of *F*^2^ > 2σ(*F*^2^) is used only for calculating *R*-factors(gt) *etc*. and is not relevant to the choice of reflections for refinement. *R*-factors based on *F*^2^ are statistically about twice as large as those based on *F*, and *R*- factors based on ALL data will be even larger.

### Fractional atomic coordinates and isotropic or equivalent isotropic displacement parameters (Å^2^)

**Table:**

	*x*	*y*	*z*	*U*_iso_*/*U*_eq_	Occ. (<1)
C1	0.1009 (10)	−0.1237 (10)	0.4794 (7)	0.025 (2)	0.50
C2	0.2646 (3)	0.1270 (3)	0.4195 (2)	0.0193 (7)	
C3	0.3138 (3)	0.1696 (3)	0.4992 (2)	0.0249 (8)	
C4	0.4438 (4)	0.1739 (3)	0.5146 (3)	0.0294 (8)	
H10	0.4765	0.2061	0.5663	0.035*	
C5	0.5256 (4)	0.1321 (3)	0.4558 (3)	0.0312 (9)	
H6	0.6121	0.1359	0.4676	0.037*	
C6	0.4771 (3)	0.0841 (3)	0.3786 (2)	0.0279 (8)	
H15	0.5311	0.0537	0.3389	0.033*	
C7	0.3484 (3)	0.0814 (3)	0.3608 (2)	0.0230 (7)	
H9	0.3168	0.0488	0.3090	0.028*	
C8	0.2326 (4)	0.2095 (4)	0.5707 (2)	0.0345 (9)	
H24A	0.1527	0.2374	0.5467	0.052*	
H24B	0.2736	0.2747	0.6024	0.052*	
H24C	0.2198	0.1418	0.6083	0.052*	
C9	0.0342 (3)	0.2733 (3)	0.3794 (2)	0.0211 (7)	
C10	0.1102 (4)	0.3733 (3)	0.4027 (2)	0.0263 (8)	
H22	0.1910	0.3603	0.4266	0.032*	
C11	0.0664 (4)	0.4919 (3)	0.3905 (3)	0.0327 (9)	
H18	0.1174	0.5581	0.4062	0.039*	
C12	−0.0537 (4)	0.5105 (3)	0.3547 (3)	0.0362 (10)	
H4	−0.0836	0.5897	0.3462	0.043*	
C13	−0.1299 (4)	0.4121 (4)	0.3314 (3)	0.0319 (9)	
H19	−0.2102	0.4264	0.3070	0.038*	
C14	−0.0886 (3)	0.2918 (3)	0.3437 (2)	0.0238 (7)	
C15	−0.1760 (3)	0.1889 (4)	0.3142 (2)	0.0285 (8)	
H14A	−0.1528	0.1600	0.2592	0.043*	
H14B	−0.2611	0.2184	0.3097	0.043*	
H14C	−0.1695	0.1233	0.3550	0.043*	
C16	0.0791 (3)	0.0561 (3)	0.2837 (2)	0.0188 (7)	
C17	0.1169 (3)	0.1212 (3)	0.2114 (2)	0.0222 (7)	
C18	0.0976 (3)	0.0660 (4)	0.1308 (2)	0.0271 (8)	
H23	0.1215	0.1074	0.0822	0.033*	
C19	0.0436 (4)	−0.0488 (4)	0.1218 (2)	0.0305 (8)	
H17	0.0326	−0.0838	0.0676	0.037*	
C20	0.0061 (3)	−0.1115 (3)	0.1923 (2)	0.0286 (8)	
H13	−0.0311	−0.1880	0.1858	0.034*	
C21	0.0239 (3)	−0.0601 (3)	0.2729 (2)	0.0235 (7)	
H5	−0.0008	−0.1029	0.3206	0.028*	
C22	0.1748 (4)	0.2474 (3)	0.2150 (2)	0.0273 (8)	
H16A	0.2179	0.2619	0.1635	0.041*	
H16B	0.2334	0.2529	0.2638	0.041*	
H16C	0.1098	0.3073	0.2200	0.041*	
O1	0.1652 (12)	−0.2035 (10)	0.4677 (9)	0.030 (3)	0.50
P1	0.09397 (8)	0.11591 (8)	0.39409 (6)	0.0182 (2)	
Cl1	0.1376 (3)	−0.1671 (3)	0.4666 (2)	0.0226 (7)	0.50
Rh1	0.0000	0.0000	0.5000	0.01825 (13)	

### Atomic displacement parameters (Å^2^)

**Table:**

	*U*^11^	*U*^22^	*U*^33^	*U*^12^	*U*^13^	*U*^23^
C1	0.029 (6)	0.018 (6)	0.027 (5)	−0.004 (4)	0.001 (4)	−0.004 (4)
C2	0.0171 (15)	0.0122 (15)	0.0287 (17)	−0.0030 (12)	0.0015 (13)	0.0071 (13)
C3	0.0302 (19)	0.0125 (16)	0.0319 (19)	−0.0026 (14)	0.0020 (15)	0.0026 (14)
C4	0.0285 (19)	0.0198 (19)	0.039 (2)	−0.0063 (15)	−0.0085 (16)	0.0042 (16)
C5	0.0226 (18)	0.023 (2)	0.048 (2)	−0.0048 (15)	−0.0030 (16)	0.0143 (17)
C6	0.0220 (17)	0.0217 (19)	0.040 (2)	0.0005 (14)	0.0065 (15)	0.0088 (16)
C7	0.0245 (17)	0.0132 (16)	0.0314 (19)	−0.0003 (13)	0.0024 (14)	0.0032 (14)
C8	0.037 (2)	0.037 (2)	0.029 (2)	−0.0065 (18)	0.0011 (16)	−0.0055 (17)
C9	0.0238 (17)	0.0160 (16)	0.0244 (17)	−0.0018 (13)	0.0089 (13)	0.0005 (14)
C10	0.0303 (19)	0.0183 (18)	0.0309 (19)	−0.0033 (15)	0.0083 (15)	−0.0002 (15)
C11	0.041 (2)	0.0162 (18)	0.042 (2)	−0.0027 (16)	0.0131 (18)	−0.0035 (16)
C12	0.045 (2)	0.020 (2)	0.046 (2)	0.0091 (17)	0.016 (2)	0.0019 (17)
C13	0.032 (2)	0.025 (2)	0.040 (2)	0.0087 (16)	0.0080 (16)	0.0019 (17)
C14	0.0231 (17)	0.0233 (18)	0.0259 (18)	0.0018 (14)	0.0097 (14)	0.0017 (14)
C15	0.0215 (17)	0.031 (2)	0.033 (2)	0.0027 (15)	0.0019 (14)	0.0020 (16)
C16	0.0165 (15)	0.0171 (17)	0.0228 (16)	0.0001 (13)	0.0013 (12)	−0.0004 (14)
C17	0.0179 (16)	0.0210 (18)	0.0279 (18)	0.0032 (13)	0.0036 (13)	0.0002 (14)
C18	0.0267 (18)	0.029 (2)	0.0256 (18)	0.0086 (16)	0.0012 (14)	0.0007 (15)
C19	0.030 (2)	0.031 (2)	0.030 (2)	0.0074 (17)	−0.0033 (15)	−0.0135 (17)
C20	0.0250 (18)	0.0208 (18)	0.040 (2)	0.0014 (15)	−0.0034 (15)	−0.0072 (16)
C21	0.0197 (16)	0.0218 (19)	0.0292 (18)	0.0033 (14)	0.0028 (13)	0.0007 (15)
C22	0.0302 (19)	0.0255 (19)	0.0268 (19)	−0.0044 (15)	0.0079 (15)	0.0041 (15)
O1	0.028 (5)	0.020 (6)	0.041 (4)	0.007 (4)	0.011 (4)	0.006 (4)
P1	0.0184 (4)	0.0132 (4)	0.0233 (4)	−0.0029 (3)	0.0037 (3)	−0.0007 (3)
Cl1	0.025 (2)	0.016 (2)	0.0269 (13)	0.0042 (13)	0.0070 (13)	0.0036 (16)
Rh1	0.01767 (19)	0.0145 (2)	0.0230 (2)	−0.00172 (14)	0.00487 (14)	0.00057 (14)

### Geometric parameters (Å, °)

**Table:**

C1—Cl1	0.654 (9)	C13—C14	1.399 (5)
C1—O1	1.131 (11)	C13—H19	0.9300
C1—Rh1	1.769 (11)	C14—C15	1.516 (5)
C2—C3	1.402 (5)	C15—H14A	0.9600
C2—C7	1.405 (5)	C15—H14B	0.9600
C2—P1	1.842 (3)	C15—H14C	0.9600
C3—C4	1.393 (5)	C16—C21	1.408 (5)
C3—C8	1.513 (5)	C16—C17	1.411 (5)
C4—C5	1.377 (6)	C16—P1	1.841 (3)
C4—H10	0.9300	C17—C18	1.401 (5)
C5—C6	1.387 (5)	C17—C22	1.513 (5)
C5—H6	0.9300	C18—C19	1.386 (6)
C6—C7	1.383 (5)	C18—H23	0.9300
C6—H15	0.9300	C19—C20	1.373 (6)
C7—H9	0.9300	C19—H17	0.9300
C8—H24A	0.9600	C20—C21	1.382 (5)
C8—H24B	0.9600	C20—H13	0.9300
C8—H24C	0.9600	C21—H5	0.9300
C9—C10	1.398 (5)	C22—H16A	0.9600
C9—C14	1.407 (5)	C22—H16B	0.9600
C9—P1	1.846 (4)	C22—H16C	0.9600
C10—C11	1.389 (5)	P1—Rh1	2.3496 (10)
C10—H22	0.9300	Cl1—Rh1	2.418 (3)
C11—C12	1.383 (7)	Rh1—C1^i^	1.769 (11)
C11—H18	0.9300	Rh1—P1^i^	2.3496 (10)
C12—C13	1.385 (6)	Rh1—Cl1^i^	2.418 (3)
C12—H4	0.9300		
			
Cl1—C1—Rh1	172.6 (14)	H14A—C15—H14C	109.5
O1—C1—Rh1	178.8 (14)	H14B—C15—H14C	109.5
C3—C2—C7	118.4 (3)	C21—C16—C17	119.7 (3)
C3—C2—P1	122.0 (3)	C21—C16—P1	116.6 (2)
C7—C2—P1	119.3 (3)	C17—C16—P1	123.7 (3)
C4—C3—C2	119.0 (3)	C18—C17—C16	117.8 (3)
C4—C3—C8	117.7 (3)	C18—C17—C22	117.8 (3)
C2—C3—C8	123.3 (3)	C16—C17—C22	124.4 (3)
C5—C4—C3	122.1 (4)	C19—C18—C17	121.4 (3)
C5—C4—H10	119.0	C19—C18—H23	119.3
C3—C4—H10	119.0	C17—C18—H23	119.3
C4—C5—C6	119.0 (3)	C20—C19—C18	120.6 (3)
C4—C5—H6	120.5	C20—C19—H17	119.7
C6—C5—H6	120.5	C18—C19—H17	119.7
C7—C6—C5	120.0 (4)	C19—C20—C21	119.6 (4)
C7—C6—H15	120.0	C19—C20—H13	120.2
C5—C6—H15	120.0	C21—C20—H13	120.2
C6—C7—C2	121.2 (3)	C20—C21—C16	120.9 (3)
C6—C7—H9	119.4	C20—C21—H5	119.6
C2—C7—H9	119.4	C16—C21—H5	119.6
C3—C8—H24A	109.5	C17—C22—H16A	109.5
C3—C8—H24B	109.5	C17—C22—H16B	109.5
H24A—C8—H24B	109.5	H16A—C22—H16B	109.5
C3—C8—H24C	109.5	C17—C22—H16C	109.5
H24A—C8—H24C	109.5	H16A—C22—H16C	109.5
H24B—C8—H24C	109.5	H16B—C22—H16C	109.5
C10—C9—C14	120.2 (3)	C16—P1—C2	105.01 (15)
C10—C9—P1	120.5 (3)	C16—P1—C9	101.78 (16)
C14—C9—P1	119.3 (3)	C2—P1—C9	107.12 (15)
C11—C10—C9	120.7 (4)	C16—P1—Rh1	116.59 (11)
C11—C10—H22	119.6	C2—P1—Rh1	109.66 (11)
C9—C10—H22	119.6	C9—P1—Rh1	115.72 (11)
C12—C11—C10	119.3 (4)	C1—Rh1—C1^i^	180.000 (2)
C12—C11—H18	120.4	C1—Rh1—P1^i^	90.0 (4)
C10—C11—H18	120.4	C1^i^—Rh1—P1^i^	90.0 (4)
C11—C12—C13	120.5 (4)	C1—Rh1—P1	90.0 (4)
C11—C12—H4	119.8	C1^i^—Rh1—P1	90.0 (4)
C13—C12—H4	119.8	P1^i^—Rh1—P1	180.0
C12—C13—C14	121.4 (4)	C1—Rh1—Cl1	2.0 (4)
C12—C13—H19	119.3	C1^i^—Rh1—Cl1	178.0 (4)
C14—C13—H19	119.3	P1^i^—Rh1—Cl1	91.66 (10)
C13—C14—C9	117.9 (3)	P1—Rh1—Cl1	88.34 (10)
C13—C14—C15	118.3 (3)	C1—Rh1—Cl1^i^	178.0 (4)
C9—C14—C15	123.7 (3)	C1^i^—Rh1—Cl1^i^	2.0 (4)
C14—C15—H14A	109.5	P1^i^—Rh1—Cl1^i^	88.34 (10)
C14—C15—H14B	109.5	P1—Rh1—Cl1^i^	91.66 (10)
H14A—C15—H14B	109.5	Cl1—Rh1—Cl1^i^	180.00 (18)
C14—C15—H14C	109.5		

Symmetry codes: (i) −*x*, −*y*, −*z*+1.
